# The Influence of Carbon Nanotubes on the Physical and Chemical Properties of Nanocomposites Based on Unsaturated Polyester Resin

**DOI:** 10.3390/nano13232981

**Published:** 2023-11-21

**Authors:** Przemysław Pączkowski, Nadiia V. Sigareva, Borys M. Gorelov, Mariia I. Terets, Yurii I. Sementsov, Mykola T. Kartel, Barbara Gawdzik

**Affiliations:** 1 Department of Polymer Chemistry, Institute of Chemical Sciences, Faculty of Chemistry, Maria Curie-Sklodowska University, Gliniana 33, 20-614 Lublin, Poland; barbara.gawdzik@umcs.pl; 2 O. O. Chuiko Institute of Surface Chemistry, National Academy of Sciences of Ukraine, 17 General Naumov Str., 03164 Kyiv, Ukraine; microft2@ukr.net (N.V.S.); bgorel@ukr.net (B.M.G.); teretsmariya@gmail.com (M.I.T.); ysementsov@ukr.net (Y.I.S.); nikar@kartel.kiev.ua (M.T.K.); 3 Ningbo Sino-Ukrainian New Materials Industrial Technologies Institute, Kechuang Building, N777 Zhongguan Road, Ningbo 315211, China; 4 Ningbo University of Technology, 201 Fenghua Road, Ningbo 315211, China

**Keywords:** carbon nanotubes, unsaturated polyester resins, nanocomposite, physical properties, chemical properties, mechanical properties, cross-linking, chemical resistance, polymer structure

## Abstract

The new actual scientific direction is in the development of different nanocomposites and the study of their medical–biological, physicochemical, and physicomechanical properties. One way to expand the functionality of nanocomposites and nanomaterials is to introduce carbon nanostructures into the polymer matrix. This study presents the properties of unsaturated polyester resins (Estromal, LERG S.A.) based on PET recyclate with multi-walled carbon nanotubes (MWCNTs): their mechanical and thermomechanical characteristics, resistance to ultraviolet radiation (UV-vis), and chemical resistance properties. The properties of the obtained materials were characterized using physical–chemical research methods. The changes in the properties of the composites for MWCNT content of 0.1, 0.3, and 0.5 wt % were determined. The results showed positive influences on the thermomechanical and mechanical properties of nanocomposites without significant deterioration of their gloss. Too much CNT added to the resin leads to heterogeneity of the composite structure.

## 1. Introduction

Unsaturated polyester resins (UPRs) consist of unsaturated polyesters and cross-linking agents.

These resins are liquid polymers that keep their solid shape after hardening and are characterized by exceptional strength and durability, as well as strength properties or aesthetic values imitating other materials [1,2]. The UPR industry is one of the most dynamically developing branches of the polymer industry. UPR can shatter and fail at the first sign of damage or defect, however, because it is a rather brittle material. For this reason, unsaturated polyester resins are mostly used in combination with reinforcing micro- and nanomaterials. They are extensively used for various applications, especially as insulation materials in many industrial sectors, and are the main object of study for many scientists [3,4,5,6,7,8,9,10,11,12]. Unsaturated polyester resins are widely used in engineering; they are used for cast insulation in electrical and radio engineering, coatings, and fiberglass used in automobile, shipbuilding, and aircraft construction. In the works [13,14,15], the authors described the properties of the products based on polyester resins.

He et al. [16] suggested dispersing graphite oxide in unsaturated polyester resin to create nanocomposites. It was found that with a small amount of filler (0.04 wt %) the fracture energy indicators increased by 55%. In [17], the authors investigated composite materials based on unsaturated polyester resin with different contents of halloysite nano-tubes with attached silica nanoparticles. Based on the unique hybrid architecture and improved properties of nanocomposites, it was shown that hybrid nanotubes can be a promising filler for highly efficient and functional polymer composites, and the manufacturing method can have consequences in the synthesis of nanohybrid materials. Also, carbon nanotubes (CNTs) were applied as fillers of polymer matrices [18]. The authors [19] studied the features of the structures, processes of melting and crystallization, and mechanical, electrical, and thermophysical characteristics of polypropylene–carbon nanotube (PP–CNT) composites. It was established that at a content of 0.5–5.0 wt %, CNTs form a continuous network of CNTs, which leads to a significant increase in compressive strength, a decrease in the amount of fracture deformation, and an increase in electrical conductivity up to five orders of magnitude, with a slight increase in thermal conductivity. Their research results concerned CNT composites with thermoplastic polymers. Pistone et al. [20] used carbon nanotubes and unsaturated polyester resins for the preparation of coatings with expected antimicrobial and mechanical durability. Their work concerned the anti-biofilm effect and biocompatibility studies on laminate, with the external layer made of CNT and UPR.

Due to their exceptional mechanical and electric properties, carbon nanotubes and graphene nanoplatelets (both sp2-carbon nanomaterials) are actively used as hybrid fillers for the development of a new generation of nanocomposite materials. Unfortunately, they exhibit poor dispersion and insufficient interfacial bonding with polymer matrices. Dispersion of CNTs in laboratory and industrial conditions is carried out by ultrasonic treatment, in vibration mills or hydrodynamic disintegrators [21,22]. The three-roll mixing method proved to be effective for viscous polymer liquids [23,24]. To obtain stable suspensions, various methods of chemical modification of the CNT surface were also used [25,26,27]. Note that CNT reinforcement has a positive effect of strengthening the matrix, but it strongly depends on the degree of dispersion of the CNT agglomerates [28,29,30,31,32,33]. The strong van der Waals attraction between nanotubes can result in the formation of large aggregates [34].

Due to their extraordinary mechanical properties, in particular, very high elastic modulus, and tensile strength, combined with a very small diameter, carbon nanotubes can reach natural frequencies of the order of THz. Based on these characteristics, they have been considered ideal candidates for several applications in high-sensitivity electromechanical devices such as resonators, sensors, and oscillators [35,36]. This important industrial applicability has prompted many researchers to focus their attention on the study of CNT vibrations [37,38,39].

In this study, carbon nanotubes are used as fillers of commercially available unsaturated polyester resin based on PET recyclate. Multi-walled carbon nanotubes (MWCNTs) were added to the recyclate as a hexane solution. After curing, the properties of the obtained nanocomposites were studied and compared to those of pure resin. Their mechanical and thermomechanical characteristics, resistance to ultraviolet radiation (UV-vis), and chemical resistance properties were studied.

## 2. Materials and Methods

### 2.1. Materials

A commercially available resin, Estromal, was used as an unsaturated polyester resin. It is an orthophthalic resin based on recycled PET (LERG S.A., Poland). The properties provided by the manufacturer are the acidic number (13.4 mg KOH g^−1^), viscosity (356 mPas at 23 °C), non-volatile content (61.2%) [PL-W-53], and reactivity factor (1.53). For curing, Luperox DHD-9 (2-butanone peroxide solution from Sigma Aldrich (St. Louis, MO, USA) and 4% polymeric cobalt solution (Department of Polymer Chemistry, Maria Curie-Sklodowska University in Lublin, Poland) were used.

Multi-walled CNTs were synthesized by the catalytic chemical vapor deposition (CCVD) method in the fluidized bed regime, which was created due to the rotation of the reactor [40]. A three-component oxide with a metal ratio of Al_2_FeMo_0.21_ was used as a catalyst. The carbon source was propylene obtained by the dehydration process of propanol. The synthesized MWCNTs have a specific surface area of ~240 m^2^ g^−1^.

### 2.2. Curing Conditions

The liquid resin and its mixtures with MWCNTs were placed in a flask and heated up to 55 °C while stirring for 2 h and vacuumed until homogeneous solutions were obtained. As curing agents, 1.1 wt % of Luperox and 0.25 wt % of 4% polymeric cobalt solution as the accelerator were used. The polymerization of the resin and its composites was carried out at room temperature and then at ~55 °C for 18 h. The filler-mass-loading C of MWCNTs in the polymer composites were 0.1, 0.3, and 0.5 wt %. The MWCNTs were added to the resin as a suspension in hexane after ultrasonic treatment.

### 2.3. Preparation of Nanocomposite Specimens

In order to study the properties of nanocomposites based on unsaturated polyester resin and MWCNTs, samples of appropriate sizes were prepared using the MFG 8037P CNC milling machine (Ergwind, Gdańsk, Poland). Specimens were obtained in the form of rectangular bars with dimensions of 80 mm × 10 mm × 4 mm and 65 mm × 10 mm × 4 mm.

### 2.4. Research Methods

#### 2.4.1. SEM of MWCNTs

Samples of carbon nanotubes were studied on the FEI Quanta 3D FEG scanning electron microscope (SEM) (Fei Company, Hillsboro, OR, USA) in high vacuum, with an accelerating voltage of 20 kV, using an ETD detector. Before the SEM analysis, the samples were coated with a thin Pd/Au layer.

#### 2.4.2. TEM of MWCNTs

The samples were ground in an agate mill to a fine powder. The obtained powder was flooded with 99.8% ethanol (POCH, Gliwice, Poland) to form a slurry and placed in an ultrasonic homogenizer for 10 s. Then, the slurry containing the sample was pipetted onto copper mesh (200 mesh/inch—200 mesh) coated with lacey Formvar stabilized with carbon and left on paper to evaporate the ethanol. Subsequently, the samples were transferred to the electron microscope. The FEI Titan G2 60–300 kV transmission electron microscope (TEM) (Hillsboro, OR, USA) equipped with a field emission gun (FEG) was used to create images of the samples. Microscopic examination of the samples was carried out at the electron beam accelerating voltage of 300 kV. TEM imaging of the microstructure of the samples was performed in bright field mode using a CCD camera as a detector.

The mapping images of the samples and determination of the distribution of elements in the samples was carried out in the STEM (scanning transmission electron microscopy) mode, collecting the energy dispersive X-ray spectroscopy (EDS) spectrum from each place corresponding to the pixels of the map, point by point. The collected maps were presented in the form of a matrix of pixels, with the color significant for the mapped element, and the intensity corresponding to the percentage content of a given element.

#### 2.4.3. Accelerated Aging Test

Accelerated aging tests were performed using a Xenon Arc Lamp Atlas Xenotest Alpha+ simulator (Chicago, IL, USA). The source of irradiation in the test chamber was a xenon lamp, emitting radiation similar to natural sunlight in the spectral irradiance from 290 to 800 nm. Accelerating aging was carried out for 1000 h according to the standard EN ISO 4892-2:2013 [41]. The parameters imitated typical outdoor weather conditions to which materials can be exposed: irradiance of 60 W m^−2^, daylight filter system, chamber temperature of 38 °C, black standard temperature of 65 °C, and relative humidity of 50%. Each sample was exposed to a dosage (NTM, Newton to meter) of about 180,000 kJ m^−2^.

#### 2.4.4. Gloss Determination

Measurements of the samples’ gloss were made using a Zehntner ZGM 1110 triple-angle gloss meter from Zehntner GmbH Testing Instruments (Sissach, Switzerland). This device operates simultaneously in one of three geometric units in which the angles 20°, 60°, and 85° correspond from a high-gloss to a matte surface (standard gloss: 20° (86.8 GU), 60° (93.4 GU), and 85° (99.7 GU)). These determinations were made according to the standard, ASTM D2457 [42]. The final result was the mean value of six measurements for the pure resin and composites with different amounts of MWCNTs before and after the accelerated aging.

#### 2.4.5. Mechanical, Thermomechanical, and Hardness Studies

Mechanical and thermomechanical properties of unsaturated polyester resin and its nanocomposites with MWCNTs were determined using a mechanical testing machine, a dynamic mechanical analyzer, and a hardness tester.

For the determination of the mechanical properties of the samples, the Zwick-Roell Z010 mechanical testing machine from ZWICK GmbH Co. (Ulm, Germany) was used. The determination was made based on a three-point bending test with a span between supports of 64 mm. The samples of 80 mm × 10 mm × 4 mm were used. The bending speed was 5 mm min^−1^. The test procedure was in accordance with the standard EN ISO 178:2019 [43].

The thermomechanical properties of the prepared composites were studied using the Q800 Dynamic Mechanical Analyzer (DMA) from TA Instruments (New Castle, NY, USA), equipped with a dual-cantilever device. Samples with the dimensions of 65 mm × 10 mm × 4 mm were tested. A temperature scanning from 0 to 200 °C was made with a constant heating rate of 3 °C min^−1^ at a sinusoidal strain with an amplitude of 10 µm and a frequency of 1 Hz. The test procedure is according to the standard [44]. From the obtained data, the glass-transition temperature, mechanical loss factor, values of storage modulus, and full width at half maximum were determined. The result was only a single measurement both before and after the accelerated aging test.

The hardness was measured based on the Shore D method using an analog hardness tester with the test stand 7206/H04 from Zwick (Ulm, Germany) at standard temperature (23 ± 2 °C). The result was obtained after 15 s. The procedure was in accordance with the EN ISO 868:2003 standard [45]. The final result was the arithmetic averaging of five individual measurements.

#### 2.4.6. Chemical Resistance

The behavior of the UPR and its nanocomposites with MWCNTs in the presence of chemical liquids was determined according to the EN ISO 175:2010 standard [46]. The samples with the dimensions 65 mm × 10 mm × 4 mm were immersed separately in airtight containers containing 50 mL of the tested liquid. They were placed in the dark at room temperature (23 ± 2 °C). The chemical resistance of the samples was tested in distilled water, 1% NaOH, acetone, and toluene. Periodically, the samples were removed from the solvents, rinsed with distilled water, and wiped gently. The mass change, ∆*m*, is determined using Equation (1) [46]:

(1) $$∆m=\frac{m_{i}-m_{0}}{m_{0}}\times 100$$

where *m*_0_ is the initial mass of the specimen, and *m_i_* is the mass of the specimen after immersion.

## 3. Results and Discussion

**Figure 1:**
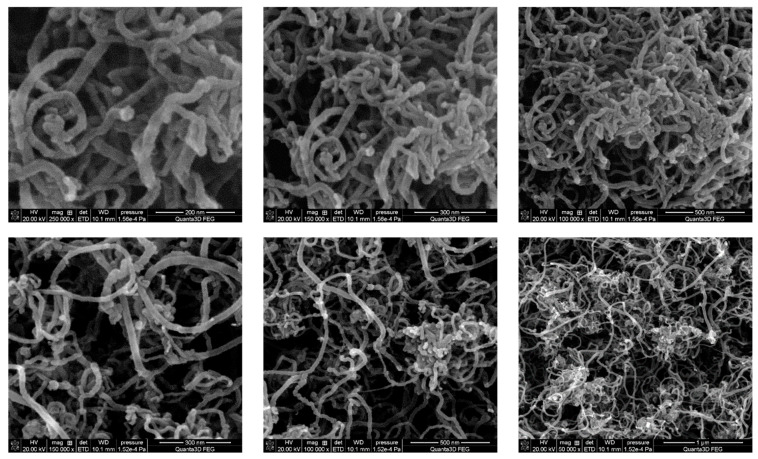
SEM images of the MWCNTs at different magnifications.

SEM photos of the used CNTs are presented in Figure 1. One can see that the studied MWCNTs look similar in all the photos. They are in the form of tangled threads with rather uniform diameters. The CNTs synthesized by the CCVD method represent agglomerates of CNTs (“balls” of intertwined “threads”). Therefore, for effective reinforcement of matrixes, in this case—polymer matrices, their preliminary deagglomeration is necessary.

Effective methods of deagglomeration and homogeneous dispersion are ultrasonic treatment in liquids with the addition of surfactants [47,48]. For viscous liquids, due to shear deformation, mixing on a three-roll mixer shows good results. In this work, as already indicated above, CNTs were added as a suspension in hexane after ultrasonic treatment.

**Figure 2:**
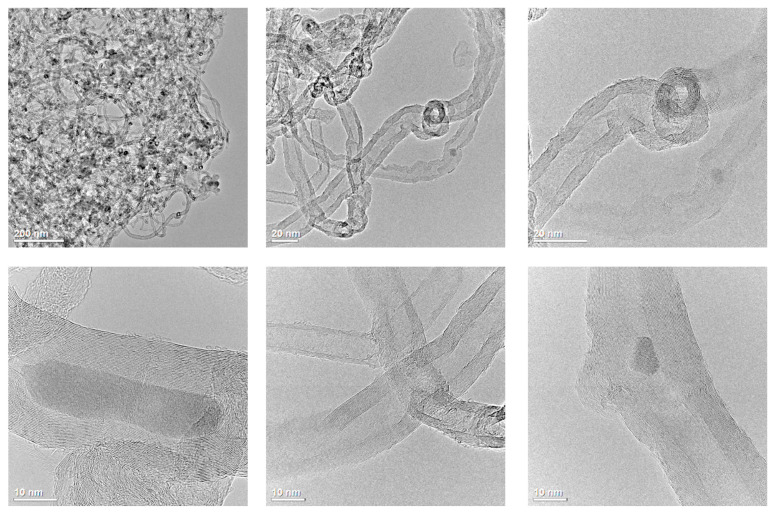
TEM images of the MWCNTs at different magnifications.

TEM images (Figure 2) confirm that the diameters of the CNTs are in the range of 10-40 nm. The cross-sections of the CNT walls are collinear over a fairly long distance, which indicates the good quality of the CNTs. In the inner cavity of the CNTs, inclusions constituting the remains of the encapsulated catalyst nanoparticles are observed.

The unique morphological properties of carbon nanotubes create an intriguing possibility of using the inner cavity to perform chemical reactions. This confinement causes the so-called “confinement effect”, which can result in different catalytic properties affecting activity, stability, and selectivity [49].

**Figure 3:**
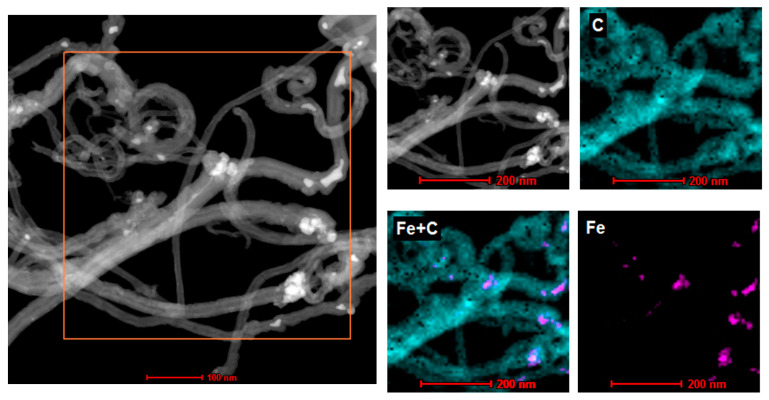
STEM-EDS mapping of MWCNTs.

STEM-EDS mapping reveals the chemical distribution in the carbon nanotubes (Figure 3). EDS microanalysis clearly confirmed the presence of carbon, the main constituent of MWCNTs. The mapping method also disclosed the existence of iron, which was the component of the catalyst used in CCVD.

In Table 1, the results of thermomechanical property studies of the UPR and its composites with MWCNTs before and after the accelerated aging test are presented.

It is assumed that carbon nanotubes retain the mechanical resilience of polyester resin in the nanocomposite. They also improve its hydrophobic character and increase the adhesion features of the coating, preventing a decrease in its stiffness due to water absorption or exposure to UV rays [50].

As can be seen in the data in Table 1, accelerated aging influences the thermomechanical properties of the resin and the corresponding composites, but in some cases, these changes were not significant. For example, in all the series after UV, the values of the storage modulus at 20 °C were higher, whereas at 180 °C, they were insignificantly lower than those of the untreated samples.

The mechanical damping coefficient, or *tan δ*, represents the ratio of the viscous to elastic response of a viscoelastic material. This parameter is useful for determining the occurrence of molecular mobility transitions, such as the glass transition temperature. The values of the mechanical loss factor ($tan\;\delta_{max}$) of the pure resin increased after accelerated aging, while for the CNT composites, it decreased. Both in the resin and its composites, the glass transition temperatures, $T_{g}$, increased after UV treatment. The greatest increase in $T_{g}$ can be observed in the pure resin. On the other hand, there is no significant effect of aging on the *FWHM* values. A slight decrease in the *FWHM* values in pure resin and composite with a 0.1% addition of CNT indicates an increase in the homogeneity of the systems, but in the sample with a 0.5% addition, the homogeneity is disturbed.

Thus, the loading with CNTs both into non-irradiated and UV-treated polyester resin samples leads to a non-monotonic change in the storage modulus value. The *E*′ value for 20 °C reduces at low nanotube contents and increases with growing filling. In addition, although the UV treatment effect improves the storage modulus value, the impact of nanotubes on the modulus behavior mitigates in the composites. Thus, after loading 0.1% CNTs into non-irradiated samples, the *E*′(20 °C) value drops by 2.4%, whereas in the UV-treated samples, the *E*′(20 °C) variation is 1.3%. As the filling increases to 0.5% CNTs in the non-irradiated samples, the *E*′ increase is 7.7%, and in the irradiated samples after the influence of UV treatment, the rise in *E*′ is 2.4%. In addition, the heat treatment of the original and UV-treated samples at 180 °C, when the thermal desorption of weakly bound, loose atomic fragments and absorbed water occurs [7], gives rise to an increase in the free volume and the *E*′(180 °C) value reduction in all the studied samples (Table 1).

A decrease in $T_{g}$ with an increase in CNT content was observed for both samples before and after UV treatment. This can be explained by the lower degree of cure achieved with the CNT content increasing, due to the dominance of the UV shielding effect, leading to a deterioration of the mechanical properties [51].

Table 2 shows the hardness of the resin and its composites after aging in the presence of UV and also after immersion in aggressive solvents. It was necessary to check which factor had a greater impact on the properties of the resin and CNT composites. It turned out that the hardness of the resin insignificantly increases after UV irradiation, as the UV light causes additional cross-linking. The samples containing CNT show higher hardness than the resin, but no significant changes in their values are observed after the UV test. After immersion in solvents, the hardness of the resin significantly decreases due to its partial degradation by the absorbed solvents, causing a loosening of bonds or hydrolysis. This effect is especially visible for the samples immersed in water and sodium hydroxide solution. On the other hand, in the samples containing CNTs, a relatively small decrease in hardness was observed, with the greatest change observed in the samples immersed in water.

The results of mechanical studies of the resin and its composites with MWCNTs are presented in Table 3. From these data, one can see that for the samples containing nanotubes, the values of flexural strength and flexural strain at break modulus are higher than those for the starting resin. In addition, it can be seen that their values increase for the samples containing 0.1% CNT and then decrease for samples with 0.5% content of nanotubes. In contrast, flexural modulus increased for a sample with 0.1% CNT and then decreased for a sample with 0.5% CNT. The latter value is lower than that of the resin matrix. This means that the addition of 0.1% CNT creates a stable, homogeneous structure of the composite, while the addition of 0.5% is too great and causes the formation of composite heterogeneity.

These results clearly indicate a positive reinforcing effect of a small amount of MWCNTs on the mechanical properties of nanocomposites. The increase in mechanical properties results from the solvent dispersion of MWCNTs and their good adhesion in the matrix. The improvement in mechanical properties results not only from the good dispersion and adhesion of MWCNTs in the resin matrix but also from the formation of chemical bonds between the MWCNTs and the UPR chains. Moreover, the higher elongations of the nanocomposites indicate that the MWCNTs compensate for the larger plastic deformations of the nanocomposites [52,53].

With a higher content of nanotubes (0.5 wt %), a large number of agglomerations are observed, which act as stress concentrations and reduce the strength of the nanocomposites, thus deteriorating their mechanical properties [54].

Table 4 presents the results of gloss measurements. Gloss is defined as the specular reflection ability of the material surface under a particular standard source and at a certain angle of incidence. This is an important parameter characterizing the surface optical properties of different materials. From the data in Table 4, one can see that before aging, all the obtained composites can be treated as high-gloss materials. Their values with 60° geometry, which is typically used, are much greater than 70 GU. For very high gloss materials such as unsaturated polyester resins, an angle of 20° is recommended. For all the samples, the deterioration in gloss is visible with the MWCNT content increase. The same tendency is observed for measurements at 20° geometry. However, the samples with MWCNTs still have gloss above 80 GU. This indicates that the addition of MWCNTs to the resin (samples with 0.1, 0.3, and 0.5%) allows for obtaining high-gloss materials.

**Figure 4:**
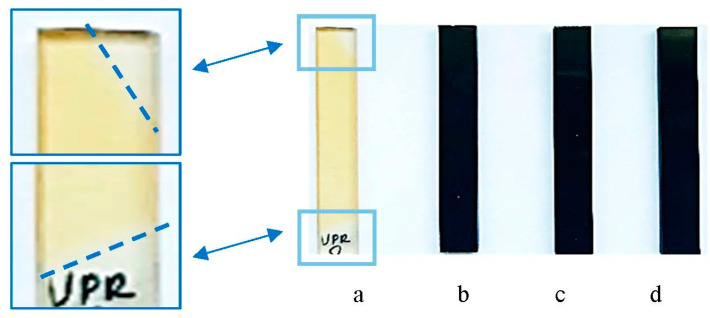
Images of the pure UPR (**a**) and its nanocomposites with 0.1, 0.3, and 0.5 wt % of MWCNTs (**b**–**d**) after UV irradiation.

**Figure 5:**
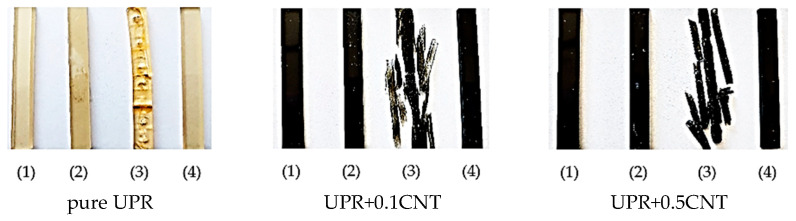
Images of the pure UPR and its nanocomposites after immersion test in distilled water (1), alkali solution (2), acetone (3), and toluene (4).

The influence of accelerated aging and the impact of aggressive solvents on the studied composites is also visible in Figure 4 and Figure 5.

In the case of irradiation with UV radiation (Figure 4), no changes in the appearance of the samples with nanotubes were observed due to their black color (Figure 4b–d), in contrast to the yellowing of the pure resin sample (Figure 4a). As can be seen in the enlarged fragments of the pure UPR specimen, due to covering and non-exposure, the parts remained unchanged and did not turn yellow.

However, it can be seen that immersion in acetone caused the destruction of the resin structure, and thus its composites with MWCNT disintegrated (Figure 5(3)). The 1% NaOH solution also had an insignificant effect on the structure of the resin. There was a slight swelling of the UPR sample, but this did not affect the appearance of the composite samples. It also turned out that both the resin and its composites are resistant to the effects of water and toluene.

**Figure 6:**
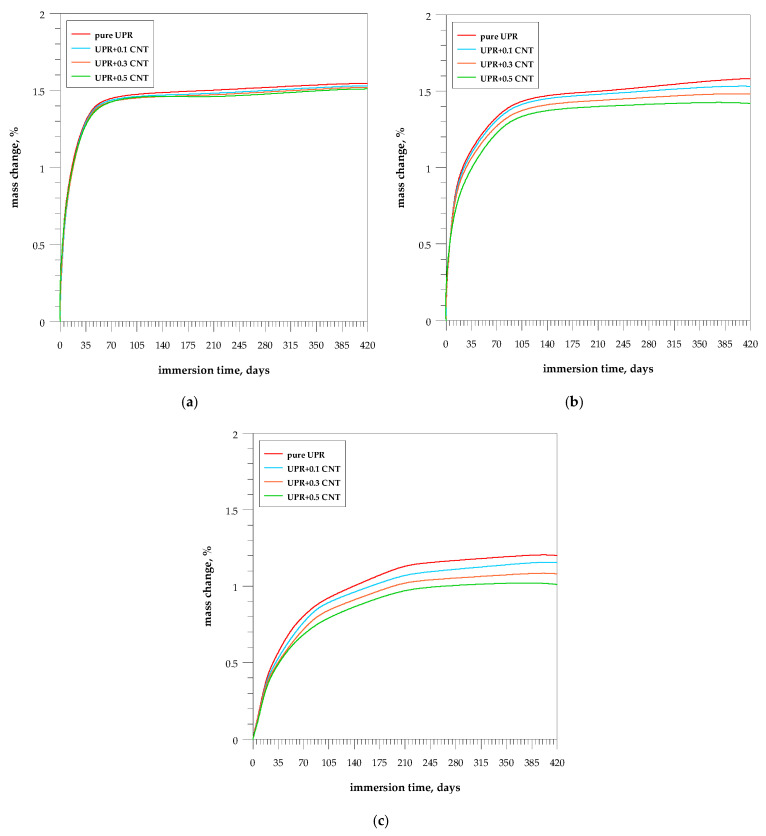
Effect of the chemical resistance of the UPR nanocomposites with MWCNTs during immersion test in (**a**) distilled water; (**b**) 1% NaOH; and (**c**) toluene.

The effect of the CNT addition on the absorption of solvents by samples over time is shown in Figure 6. The results refer to 420-day studies. The studies show that in distilled water, both pure resin and its composites increase their mass by about 1.5% after about 70 days. In 1% NaOH, the resin mass increases very slowly throughout the study, reaching 1.5% after 420 days, while the composites reach a plateau after about 105 days, with the weight of the absorbed solution decreasing as the CNT content of the composites increases.

The samples show the lowest absorbency in toluene. The mass gain for CNT composites is lower than that for pure resin and does not exceed 1% for composite with 0.5% CNT addition, but equilibrium is established after about 200 days.

Nanocarbon relies almost entirely on aromatic, non-polar, and hydrophobic tubes, so interaction with extremely polar molecules such as water is very weak.

Due to the incorporation of MWCNTs, the free volume of the solid molecular polyester could be changed by creating additional voids in the MWCNT agglomerates and in the interfaces between the MWCNTs and the polyester. Moreover, it is known that CNTs can affect the curing behavior of polyester and thus lead to a change in molecular packing and segmental mobility that directly affects the free volume [55].

The addition of carbon nanotubes to the UPR delays the cure reaction. Based on the work on nanofibers by Monti et al., similar conclusions can be drawn [56]. The nanotube–matrix interactions should be taken into account, whereas in unsaturated polyesters, the free radical scavenging activity of the carbon-based fillers can be considered the main mechanism. The CNTs can entrap the free radicals generated by the initiator within their aromatic walls due to their huge surface area. As a result, the styrene cannot react fully with the polyester molecules and is forced to self-polymerize.

Since the amount of MWCNTs added has no noticeable effect on water uptake in this study, the results obtained may be due to a change in curing behavior [57]. Due to the atomically smooth hydrophobic graphitic surface and nanoscale confinement, the nanofiller also can act as a diffusion barrier.

## 4. Conclusions

The presented results show that the addition of CNT to unsaturated polyester resin has a positive effect on the obtained composite properties. It was observed that the CNT-containing samples had lower water and alkali uptake compared to the pure resin. The results of mechanical and thermomechanical studies of the UPR/CNT composites before and after the UV test show that composites with 0.1% MWCNT form homogeneous structures. The addition of 0.5% CNT caused an increase in the composites’ heterogeneity. The values of mechanical loss factor for the pure resin increased after accelerated aging, while for CNT composites, it decreased. Glass transition temperatures, $T_{g}$, increased after UV treatment, but the greatest effect is seen for the sample with 0.1% CNT addition. The samples containing CNT show higher hardness than the resin, but no significant changes in their values are observed after the UV test. It is understandable that with the increase in the concentration of CNT in the composite samples, the gloss decreased, but still the composites obtained should be classified as high-gloss materials.

## Figures and Tables

**Table 1:** Thermomechanical data for the UPR/CNT composites before and after UV test.

Sample	$E^{\prime }$ ^1^				${tan\;\delta }_{max}$ ^2^		FWHM (°C) ^3^		$T_{g}$ (°C) ^4^	
	$E^{\prime }$(20 °C) (GPa)		$E^{\prime }$(180 °C) (MPa)						$\mathrm{from}\;{tan\;\delta }_{max}$	
	before	after	before	after	before	after	before	after	before	after
pure UPR	2.799	3.079	18.90	18.89	0.4850	0.4995	38.86	38.23	126.6	129.9
UPR + 0.1CNT	2.741	3.039	19.63	18.61	0.4895	0.4875	40.02	39.36	122.6	128.3
UPR + 0.5CNT	2.970	3.112	21.28	20.78	0.4721	0.4679	37.54	39.63	125.9	127.9

^1^ Storage modulus, glassy and rubbery; ^2^ mechanical loss factor; ^3^ full width at half maximum; ^4^ glass-transition temperature.

**Table 2:** Shore hardness of the UPR and composites with CNT (before and after accelerated aging or immersion tests).

Sample	Shore Hardness (ShD)				
	before Aging Test	after UV Irradiation	after Water Immersion	after Alkali Immersion	after Toluene Immersion
pure UPR	81.6	81.9	78.4	77.0	80.4
UPR + 0.1CNT	82.0	81.7	79.2	80.6	81.2
UPR + 0.5CNT	82.4	82.3	80.0	81.2	81.8

**Table 3:** Mechanical data of the UPR and composites with MWCNTs (mean ± SD; n = 3).

Sample	$E_{f}$ (GPa) ^1^	$\varepsilon_{f}$ (%) ^2^	$\sigma_{f}$ (MPa) ^3^
pure UPR	3.32 ± 0.02	3.46 ± 0.02	107.69 ± 3.68
UPR + 0.1CNT	3.49 ± 0.02	4.23 ± 0.03	127.97 ± 3.83
UPR + 0.5CNT	3.24 ± 0.02	3.47 ± 0.03	113.27 ± 3.39

^1^ Flexural modulus; ^2^ flexural strain at break; ^3^ flexural strength.

**Table 4:** Gloss measurement data of the studied composites (before and after immersion test).

Sample	Gloss (GU)											
	20°	60°	85°	20°	60°	85°	20°	60°	85°	20°	60°	85°
	before Immersion Test			Water Immersion			Alkali Immersion			Toluene Immersion		
pure UPR	134.5	129.3	102.5	136.2	128.2	101.0	55.9	89.0	94.9	87.8	115.0	98.6
UPR + 0.1CNT	98.0	101.5	99.8	97.8	100.1	97.9	56.7	90.7	98.5	87.7	96.2	96.1
UPR + 0.5CNT	82.2	96.1	97.8	95.0	99.1	99.3	26.9	65.1	82.6	87.9	96.2	95.7

## Data Availability

The data presented in this study are available on request from the corresponding author.
